# Sowing Density: A Neglected Factor Fundamentally Affecting Root Distribution and Biomass Allocation of Field Grown Spring Barley (*Hordeum Vulgare* L.)

**DOI:** 10.3389/fpls.2016.00944

**Published:** 2016-06-28

**Authors:** Vera L. Hecht, Vicky M. Temperton, Kerstin A. Nagel, Uwe Rascher, Johannes A. Postma

**Affiliations:** ^1^Plant Sciences, Institute of Bio- and Geosciences, Forschungszentrum Jülich GmbHJülich, Germany; ^2^Institute of Ecology, Leuphana University of LüneburgLüneburg, Germany

**Keywords:** biomass allocation, field, root architecture, root length density, root morphology

## Abstract

Studies on the function of root traits and the genetic variation in these traits are often conducted under controlled conditions using individual potted plants. Little is known about root growth under field conditions and how root traits are affected by agronomic practices in particular sowing density. We hypothesized that with increasing sowing density, root length density (root length per soil volume, cm cm^−3^) increases in the topsoil as well as specific root length (root length per root dry weight, cm g^−1^) due to greater investment in fine roots. Therefore, we studied two spring barley cultivars at ten different sowing densities (24–340 seeds m^−2^) in 2 consecutive years in a clay loam field in Germany and established sowing density dose-response curves for several root and shoot traits. We took soil cores for measuring roots up to a depth of 60 cm in and between plant rows (inter-row distance 21 cm). Root length density increased with increasing sowing density and was greatest in the plant row in the topsoil (0–10 cm). Greater sowing density increased specific root length partly through greater production of fine roots in the topsoil. Rooting depth (D50) of the major root axes (root diameter class 0.4–1.0 mm) was not affected. Root mass fraction decreased, while stem mass fraction increased with sowing density and over time. Leaf mass fraction was constant over sowing density but greater leaf area was realized through increased specific leaf area. Considering fertilization, we assume that light competition caused plants to grow more shoot mass at the cost of investment into roots, which is partly compensated by increased specific root length and shallow rooting. Increased biomass per area with greater densities suggest that density increases the efficiency of the cropping system, however, declines in harvest index at densities over 230 plants m^−2^ suggest that this efficiency did not translate into greater yield. We conclude that plant density is a modifier of root architecture and that root traits and their utility in breeding for greater productivity have to be understood in the context of high sowing densities.

## Introduction

Most of our knowledge of root system architecture and traits derives from controlled experiments in which solitary plants are grown in pots (Løes and Gahoonia, 2004; Lotfollahi, 2010), whereas data from field conditions are still relatively rare. This is partly because roots are difficult to access and evaluate in the field and relatively intensive sampling is needed to compensate for large variation caused by soil heterogeneity and other factors. Various root traits may enhance plant productivity by increasing drought tolerance and/or nutrient acquisition efficiency and may thereby be targeted by breeders (Postma and Lynch, 2011; Comas et al., 2013; Raza et al., 2014; Svačina et al., 2014; Heřmanská et al., 2015). The feasibility and relevance, however, of targeting root traits in breeding programs is still questioned as (a) high plasticity may cause traits to have low inheritance values, (b) phenotyping for root traits often has low throughput and low precision complicating the selection process, and (c) the function of the traits need to be understood under field conditions (Cobb et al., 2013; Fiorani and Schurr, 2013; Araus and Cairns, 2014; Kuijken et al., 2015; Paez-Garcia et al., 2015). Farmers, grow crops at relatively high target sowing densities in order to maximize yield. If the controlled environment studies are to have any relevance to breeding and agronomy, it is of great importance to know how sowing density influences root architecture, what traits are density-independent and to what extent density influences the root ideotype for nutrient and water acquisition. So far, little is known about the responses of roots to changing sowing densities, as most studies which deal with sowing density focus on the aboveground part of the plant. Further, sowing density can be easily changed by the farmer, so it is important to know how management influences root traits and thereby the agroecology of the crop.

Aboveground, increasing sowing density is known to decrease individual shoot biomass (Harper, 1977). In barley, increasing sowing density reduces tiller formation (Kamel, 1959; Munir, 2002; Turk et al., 2003; Soleymani et al., 2011). At very high densities, the smallest number of individual tillers was observed and in some cases plants may even not have survived, i.e., self-thinning occurs (Harper, 1977). Decreasing plant size may have direct consequences for the size-dependent root architectural traits, for example maximum rooting depth. However, due to growth regulatory mechanisms in response to plant density, plants may compensate by changing both their biomass allocation, architecture and morphology. Some of these changes are well-described for aboveground plant tissues. Etiolation responses to plant density, for example, can cause crop height to increase with increasing sowing density [for barley e.g., Turk et al. (2003), and Soleymani et al. (2011)], despite individual plants having reduced aboveground biomass. Similarly, maximum rooting depth might not simply be a function of plant size, and might become deeper, rather than shallower at higher densities. In either case, maximum rooting depth is known to be of critical importance for water acquisition and recovery of deep nitrate (Thorup-Kristensen, 2001, 2006; Lynch, 2013) which underlines the importance of knowing how sowing density may influence these traits.

While individual shoot biomass decreases with density, total biomass per area and grain yield increase with sowing density (for spring barley e.g., Kamel, 1959; Singh and Singh, 1981; Munir, 2002; Turk et al., 2003; Farnia et al., 2014), leveling-off at very high sowing densities (Singh and Singh, 1981; Farnia et al., 2014). Reviewing this response, Weiner and Freckleton (2010) concluded that total biomass on a given area was linearly proportional to plant density up to a critical plant or stand density beyond which total biomass per area does not increase (final constant yield). Changes in biomass allocation and individual plant morphology, however, may still occur. Plants become elongated (e.g., Turk et al., 2003; Soleymani et al., 2011) and allocate more to stems than to leaves (Poorter et al., 2012). Eventually, the biomass allocation to reproduction may be reduced as well, causing a lower harvest index at very high sowing densities (Weiner and Freckleton (2010), or *Trifolium incarnatum* Weiner (1980), for barley e.g., Farnia et al. (2014). These responses to density, however, have partly been bred out of the modern grain cultivars, as these cultivars (in contrast to older cultivars and land races) stay short, and maintain a high harvest index at high densities (Lee et al., 1989; Hammer et al., 2009; Soleymani et al., 2011; York et al., 2015). It is unclear how biomass partitioning to roots is affected by plant density, and whether breeding for short straw varieties and high harvest index has affected biomass partitioning to roots. Chloupek et al. (2006) observed that the root system size of semi-dwarf genotypes was significantly greater than of non-semi-dwarf controls, but Wojciechowski et al. (2009) found no effect of dwarfing genes on root elongation in either field or in soil-filled columns. Since much root phenotyping is done under non-competitive growth conditions, we asked to what extent and in what way biomass partitioning to roots of modern barley cultivars is affected by sowing density.

As we are not aware of any reports (except Kamel, 1959) in the literature of how plant density may alter barley root system architecture, we draw on a limited set of reports from other species that we found in the literature. Archer and Strauss (1985) observed steeper and greater root length densities (RLD) in denser stands of grapevine. Similarly, Azam-Ali et al. (1984) observed faster and deeper root growth at higher sowing densities of pearl millet. Manschadi et al. (1997) found an increase in RLD with increasing sowing density and over time for faba beans, although for the high sowing densities, RLD decreased after pod setting. Several studies of RLD in high density stands report high RLDs in the topsoil (for example Tardieu, 1988; Mommer et al., 2010; Kucbel et al., 2011; for example Chen et al., 2013; Ravenek et al., 2014). However, many of these studies do not contain low density controls, and most plants might forage the topsoil with greater intensity simply as the topsoil has generally greater nutrient availability (Jobbágy and Jackson, 2001; Kahle et al., 2010). Apparently, most stands forage the topsoil with a greater intensity and hence, we hypothesize that high sowing density will increase RLD in the topsoil. Whether topsoil foraging is a desirable trait in agriculture is still under discussion and probably depends strongly on the soil environment (Thorup-Kristensen, 2001; Dunbabin et al., 2003; Ho et al., 2005; Zhu et al., 2005; Lynch, 2013).

We ask if roots and shoots respond in a similar way to sowing density and how sowing density would influence plant growth, crop production (final grain yield) and biomass allocation. To address these questions, we set up two sowing density field experiments with spring barley over 2 consecutive years. We hypothesized that with increasing sowing density, root length density (root length per soil volume) will increase as well as specific root length (root length per root dry weight) due to relatively greater investment in fine roots. We expected these increases in root length density to be greater in the topsoil.

## Material and methods

To study the effect of sowing density on root and shoot growth and yield, we conducted sowing density experiments with spring barley in a field in Germany over 2 consecutive years. We took soil cores to investigate root length distribution around the time of flowering of two barley cultivars grown at ten different sowing densities.

### Plant material

We grew two German malting spring barley (*Hordeum vulgare* L.) cultivars “Scarlett” and “Barke.” Scarlett grows shorter than Barke, however, Barke is more resistant to lodging. Scarlett ripens earlier than Barke (Lindemann et al., 2002). Barke is often used in scientific studies (Gahoonia and Nielsen, 2004; Schmalenbach and Pillen, 2009; Auškalnienė et al., 2010; Dornbusch et al., 2011; Castillo et al., 2012; Füllner et al., 2012; Alqudah and Schnurbusch, 2015).

### Field site

We conducted the experiments at Campus Klein-Altendorf (University of Bonn, Germany, 50°37′31.00″N, 6°59′20.54″E) in 2013 and 2014 on a loamy-clay silt soil (luvisol). Annual precipitation, average annual temperature and sun hours were 734.4 mm, 9.8°C and 1753 h in 2013 and 820.4 mm, 11.4°C, and 1934 h in 2014, respectively, and cumulative rainfall, thermal time (cumulative growing degree days), and cumulative photosynthetically active radiation (PAR) from sowing date until final harvest date were 285 mm, 1769.06°C, and 68.9 kWh m^−2^ in 2013 and 315 mm, 1864.45°C, and 74.9 kWh m^−2^ in 2014 (see Figure S1). Climate data were obtained from the service center of the rural area of Rhineland-Palatine (Dienstleistungszentrum Ländlicher Raum Rheinland-Pfalz)^1^ and can be found on http://www.am.rlp.de.

### Experimental design

**Figure 1:**
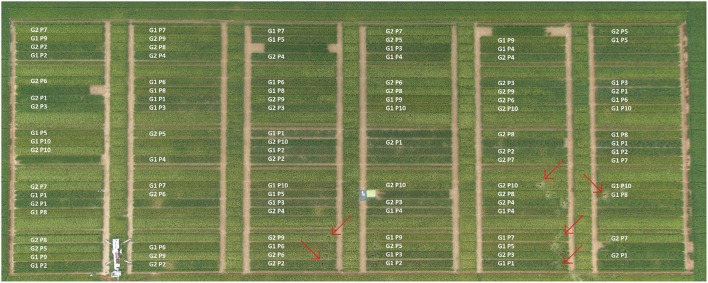
**Experimental design of 2014 at 90 DAS (1179.75°C GDD [growing degree days = average of daily maximum and minimum temperature minus base temperature (here, base temperature = 0°C), adapted according to McMaster and Wilhelm (1997)]**. G1 and G2 refer to cultivar Scarlett and Barke. P1–P10 stand for the 10 different sowing densities: 24, 31, 43, 68, 120, 140, 190, 238, 298, and 340 seeds m^−2^, respectively. Plots were 14.2 m long and 1.5 m wide. Data of the plots not used within this publication are left blank. Red arrows indicate some positions of coring. The design of 2013 can be found in Supplementary Figure S2. Picture with permission of A. Burkart.

Sowing took place on 25 April 2013 and 20 March 2014 in 1.5 × 14.2 m plots in six rows (inter-row distance of 21 cm) in a randomized nested-block design with five replicates in ten different sowing densities (24, 31, 43, 68, 120, 140, 190, 238, 298, and 340 seeds m^−2^ as sowing density 1–10, respectively (recommended sowing density for spring barley in Germany between 250 and 300 seeds m^−2^); Figure 1, Figure S2) using a Hege 95 single seed sowing machine (Hege, Waldenburg Germany). We took soil cores (9 cm in diameter, hammer: COBRA; cylinder: Eijkelkamp) in all sowing densities (in the plant row and between the plant rows, each *n* = 1) plus additional replicates in the lowest (24 seeds m^−2^), medium (120 seeds m^−2^), and highest (340 seeds m^−2^) sowing densities (2013: in the plant row, *n* = 3; 2014: only Scarlett in the plant row and between the plant row, each *n* = 4). In 2013, we sampled 60 cm deep at 48–56 DAS (stem elongation phase, BBCH 30–49). We reduced the coring depth to 40 cm deep in 2014 at 88–97 DAS (around flowering, BBCH 69–87), as the samples below 40 cm contained relatively few roots and we did not observe any treatment effects below 40 cm. Soil coring was always complemented with shoot measurements, as described below. We harvested the grain for determining the final yield on 16 August 2013 (113 DAS) and 26 July 2014 (128 DAS), respectively.

### Crop husbandry

Plants were sprayed against pathogens and insects as recommended for barley cultivation (2013: insecticide Karate Zeon at BBCH 12, herbicides Azur and Hoestar, and fungicide Capalo at BBCH 29, and fungicide Adexar and insecticide Biscaya at BBCH 59; 2014: herbicides Azur and Hoester at BBCH 13, Capalo at BBCH 30, fungicides Input and Karate Zeon at BBCH 37, fungicide Adexar at BBCH 61). Fertilization was the same for all treatments and based on soil tests; in 2013: basis fertilization P_2_O_5_: 45 kg/ha, K_2_O: 160 kg/ha, MgO: 24 kg/ha; N-application (total): 50 kg/ha; in 2014: basis fertilization P_2_O_5_: 30 kg/ha, K_2_O: 60 kg/ha, MgO: 9 kg/ha; N-application (total): 45 kg/ha. In both years, N-application was somewhat lower than recommended for spring barley to avoid lodging (recommended N-application (total): 80–120 kg/ha).

### Non-destructive measurements during growth

Approximately every 2 weeks we evaluated non-destructively three randomly chosen plants per plot. We stretched the plant to measure plant height from the plant base to the tip of the longest leaf (to the nearest 0.1 cm). We recorded the developmental stage of the plants according the so called BBCH stages (Lancashire et al., 1991). Further, we counted all tillers of an investigated plant (tiller count).

### Shoot sampling at coring

To determine shoot traits, we collected five plants of each plot at each sampling time by cutting the plants at the base (ground level) in 2013 and for sowing density 5–10 in 2014, while only three plants per plot were harvested for sowing density 1–5 in 2014 (88–97 DAS), in order to reduce the amount of sampled plant tissue. Of the harvested plants, we took three randomly chosen tillers as a sub sample, separated them into leaf sheaths and blades and photographed them to determine leaf area via segmentation based on green value. We oven dried the sub-samples and remaining shoot samples at 70° C for at least 2 days before determining their dry weight (to the nearest 0.01 g). We calculated specific leaf area (SLA) as leaf blade area over its corresponding dry weight.

### Soil coring, root washing, and sample processing

The soil cores, taken within 1 day after the harvesting of the shoots, were divided into 10 cm sections and individually packed into plastic bags and stored at 4°C until root washing. We manually washed the roots of each soil core section separately on a sieve (mesh size 500 μm) using tap water. After cleaning, we collected the roots from the sieve and stored them in 50% EtOH in 2013 and, since the handling of roots without EtOH was much easier and the time between washing and scanning was maximum 2 weeks, in tap water at 4°C in 2014, respectively, before scanning and analyzing with WinRHIZO™ (resolution 600 dpi, gray scale, manual threshold gray value 210, 20 diameter classes à 0.1 mm width). We oven-dried the scanned roots at 70°C until showing constant weight (to the nearest 0.00001 g). Similar to D95-values (depth of 95% of root length in a core) as in e.g., Lynch (2013) and Zhan et al. (2015), we calculated D50-values (median of the interpolated root length distribution, i.e., the depth in cm which divides the root length in the core into equal parts) on root length values down to 40 cm depth to be able to compare the data of the 2 years.

### Root measures

From the scans in WinRHIZO™, we obtained total root length (TRL) for each core section and calculated root length density (RLD) for each layer by dividing through the corresponding core volume. Furthermore, we calculated specific root length (SRL) for each layer separately as root dry weight (RDW) by its corresponding TRL.

### Dry weight ratios

In order to calculate the biomass fractions, we converted shoot dry weight per plant and root dry weight per volume to dry weight per area using the here described formulas. Further, we only used the root dry weights of 0–40 cm depth in order to be able to compare the data of the 2 years. Hence, we calculated total plant dry weight per area (TPDW) as

$$\begin{array}{l} TPDW=\left(\frac{shoot\;dry\;weight}{plant}*\frac{seeds}{area}\right)+RDW,\text{in g }{\text{m}}^{-2} \end{array}$$

with root dry weight per area of columns (RDW) as

$$\begin{array}{l} RDW=\frac{\frac{root\;dry\;weight}{0-40\;cm\;soil\;column}}{area\;of\;column},\text{in g }{\text{m}}^{-2} \end{array}$$

and used it to calculate root mass fraction (RMF), stem mass fraction (SMF) and leaf mass fraction (LMF) as

$$\begin{array}{l} RMF=\frac{RDW}{TPDW}, \end{array}$$

$$\begin{array}{l} SMF=\frac{\frac{stem\;dry\;weight\;of\;three\;tillers}{dry\;weight\;pf\;three\;tillers}*\frac{shoot\;dry\;weight}{plant}*sowing\;density}{TPDW}, \end{array}$$

and

$$\begin{array}{l} LMF=\frac{\frac{leaf\;dry\;weight\;of\;three\;tillers}{dry\;weight\;pf\;three\;tillers}*\frac{shoot\;dry\;weight\;}{plant}*sowing\;density}{TPDW}. \end{array}$$

### Yield determination

At final harvest, we determined the total weight of the seeds per plot (yield at harvest) and took a 100 g subsample for oven-drying at 105°C and determining seed dry matter. We corrected the seed weights for water content as follows:

$$\begin{array}{l} yield=\frac{yield\;at\;harvest\times determined\;seed\;dry\;matter}{basic\;seed\;dry\;matter}, \end{array}$$

with basis seed dry matter = 86% (Richtlinien für die Durchführung von landwirtschaflichen Wertprüfungen und Sortenversuchen, 2014)^2^.

### Statistics

We used R version 3.2.3 (R Development Core Team, 2015) to analyze all data. As the two genotypes were not statistically different from each other in response to almost all of the measured parameters (exception: plant height with Barke taller than Scarlett, RMF not affected for Barke and declining for Scarlett in 2014), we pooled them for the analysis and consider our data as more generally true, and not cultivar dependent, although genotypic differences in plant responses to sowing density may exist among other genotypes. We fitted three different models: first, we fitted linear regressions (model 1) but if it was not appropriate (*R*^2^ < 0.3), we applied linear regression on 1/y transformed data which is according to Willey and Heath (1969) for density-dependent data the most satisfactory equation (model 2), or non-linear saturating curves (Michealis-Menten kinetics) which is an inversion of model 2 and better describes saturating or asymptotic relationship between sowing density and e.g., yield (model 3; Willey and Heath, 1969). For the linear models, we used ~*a*+b^*^x+c^*^x^2^, but dropped terms if it would improve the AIC criteria (using the R function stepwise). The 1/y data transformation was fitted in the same way as the non-transformed data and used for data that seemed appropriate. For example, number of tillers per hectare was relatively linear with density, which means that the number of tillers per plant is mathematically a simple inversion with density. As responses to density are known to saturate at higher densities, linear fits do not always describe the density dose-response curves in a satisfactory way, and we fitted the nonlinear function $y=a+\frac{\left(b*x\right)}{\left(c+x\right)}$ (with *y* = measured trait, *x* = sowing density) to the data using *R*'s nls-function. Figures show model fits with 95% confidence intervals shaded in gray. Raw data can be found in the Supplementary Material.

## Results

The results of the two cultivars were in almost all measured parameters the same. We therefore merged the data of the two cultivars for the analysis and here present the results of the combined analysis. If the cultivars differed in a measured trait, we point out the difference in the corresponding section below.

### Sowing density affects tiller formation and aboveground biomass production

**Figure 2:**
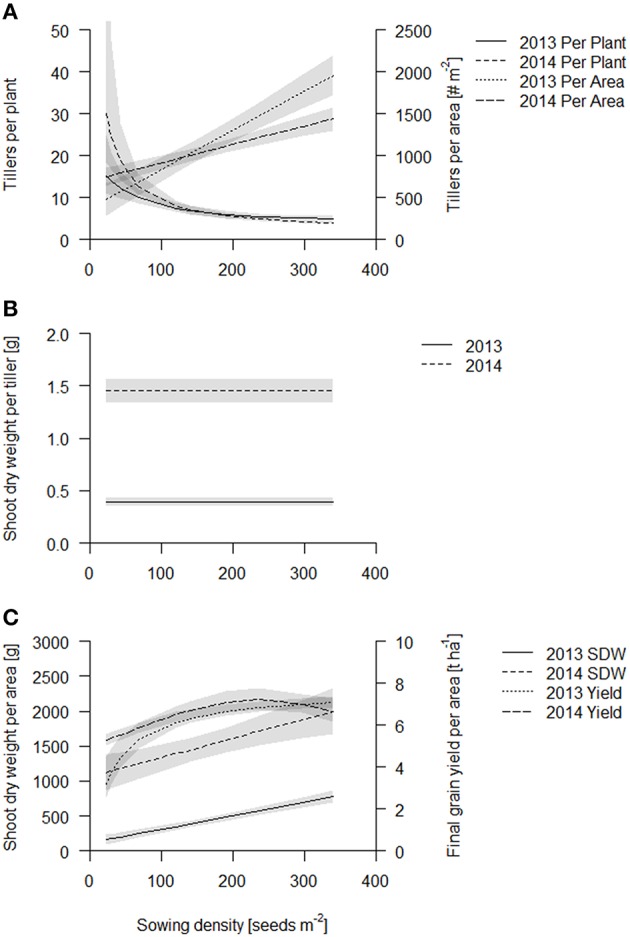
**Aboveground traits of the coring events of Scarlett and Barke in 2013 (solid line) and 2014 (dashed line)**. Data are presented as best fits with 95% confidence interval (gray). For equations, *R*^2^ and *p*-values see Table S1. **(A)** Tillers per plant and tillers per area; **(B)** Shoot dry weight per tiller; **(C)** Shoot dry weight per area and final grain yield.

Tiller count per plant was constant across all densities during the first 4 weeks of crop establishment (see Figure S3). At 4 weeks after sowing, plants had 2–5 tillers and this number did not further increase in the highest sowing densities (≥140 seeds m^−2^) over time. This tillering arrest was observed in both years (see Figure S3). For the lower sowing densities (24–120 seeds m^−2^), tillering was arrested at later times, namely the earlier the higher the sowing density, and the rate of tiller formation was negatively correlated with sowing density. Consequently, the fit of tillers per plant at the coring event declined exponentially (Figure 2A, see Table S1). This decline from lowest to highest sowing density was about 5–6 times in 2013 (during stem elongation) and 6–7 times in 2014 (during grain filling). This relative decline in tiller number per plant was not as strong as the 14-fold increase in sowing density, so that the number of tillers per area increased with sowing density in both years, and the increase was approximately linear at the coring events. In 2013, the intercept of the linear fit was lower and the slope was steeper, possibly reflecting the earlier sampling time, however, yield was also more sensitive to sowing density in 2013 (see below). Surprisingly, at high densities, the number of tillers per m^2^ was less in 2014 than in 2013. This might be simply year to year variation, but could also reflect senescence of smaller tillers or plants in the highest densities and thus a form of self-thinning. True self-thinning, that is mortality of the plant, could present an error in our estimation of shoot biomass and tiller counts per m^2^, as we took our measures on individual plants. However, we took great care that we only sampled locations where all plants and neighboring plants were present.

The shoot dry weight per tiller stayed constant over sowing density and was 0.33 and 1.4 g per tiller in 2013 and 2014 respectively (Figure 2B, Table S1). Consequently, just as the tiller counts per area, shoot dry weight per area increased linearly in both years (Figure 2C, see also Table S1, Figure S4). Both fits of shoot dry weight per area had about the same slope, however, the intercept was significantly greater in 2014, in accordance with the later sampling time point (Figure 2C, Table S1). The increase of shoot dry weight per area from lowest to highest sowing density was five times in 2013 and two times in 2014. Biomass production is thought to be closely related to light capture, which at later stages, when the canopies at all densities have closed (full canopy closure at 62 DAS, data published elsewhere; Burkart et al., in preparation), is independent of sowing density. But absolute differences in biomass production, gained during earlier stages of development, are maintained.

In both years, greatest grain yield (~7.5 t ha^−1^) was obtained at a sowing density of 230 seeds m^−2^ and declined slightly at higher sowing densities (Figure 2C, Table S1). Grain yield was more sensitive to low sowing densities in 2013 than in 2014, so that the increase in yield from the lowest to the highest sowing density was about two times in 2013 and 1.5 times in 2014. Grain yield per area was the same for the two cultivars except in lower sowing densities (below 68 seeds per m^2^), where Barke had somewhat lower values than Scarlett (see Figure S5). The grain yield suggests that early gains in biomass production at the high densities might not translate into grain yield.

In summary, during early crop development sowing density has little effect on individual plant development, and consequently the tiller count per area and the biomass production per area is greater at greater densities. However, as the crop develops, individual plants responded to the density treatment by reducing tiller formation but not the growth rate of the individual tillers. Eventually the crop canopy closes and biomass production per tiller and per area becomes less sensitive to sowing density. Early gains in biomass in the higher sowing densities also translate into increased yield, but at sowing densities greater than 230 plants m^−2^ yield stabilizes or declines slightly.

### Sowing density affects biomass partitioning

**Figure 3:**
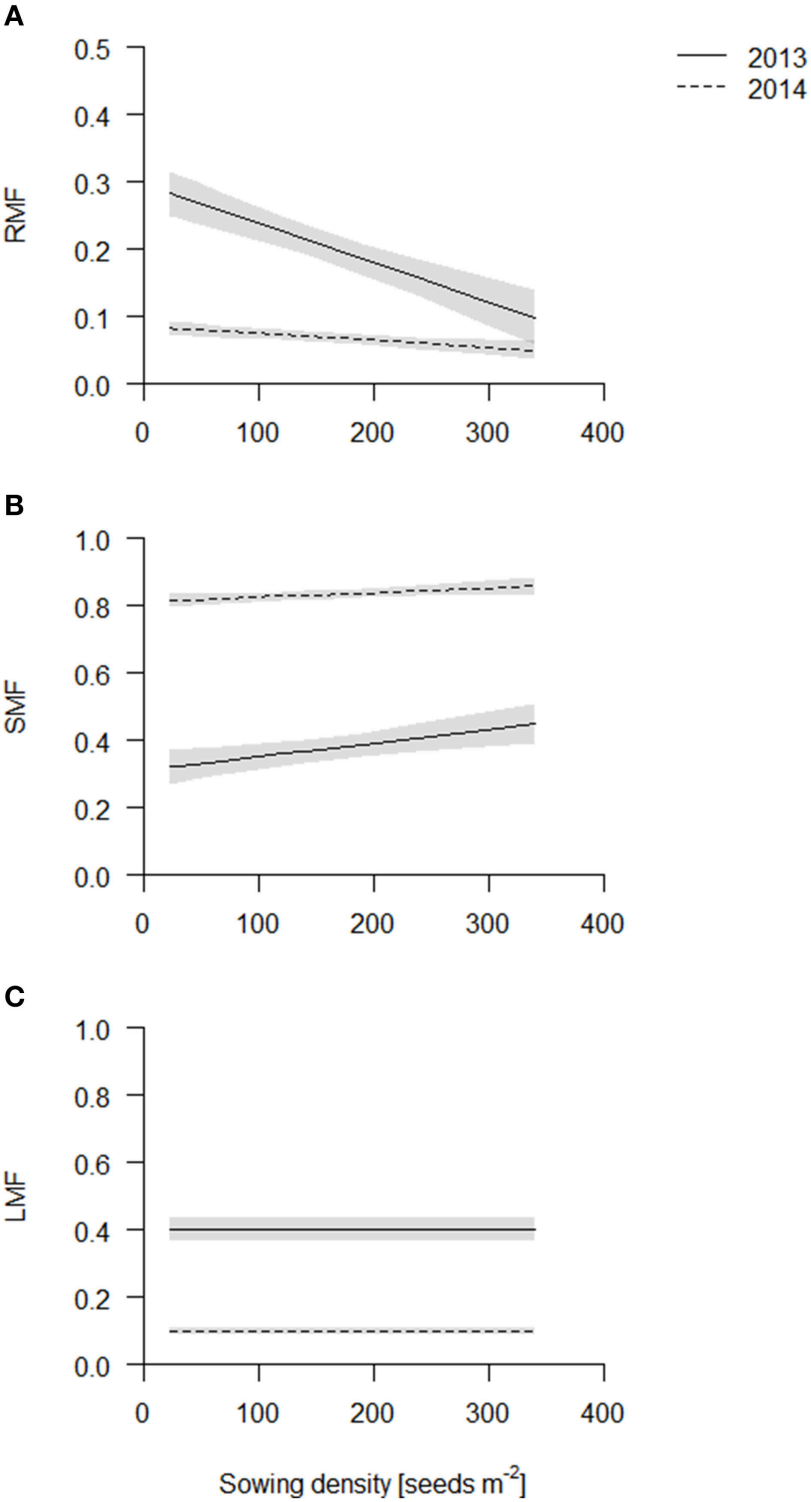
**Biomass partitioning of the coring in 2013 (solid line) and 2014 (dashed line)**. Data are presented as best fits with 95% confidence interval (gray). For equations, *R*^2^ and *p*-values see Table S2. **(A)** Root mass fraction (RMF); **(B)** stem mass fraction (SMF); **(C)** leaf mass fraction (LMF).

The root dry weight in the cores increased with sowing density, but to a lesser degree compared to the shoot dry weight and consequently root mass fraction (=root dry weight per area up to 40 cm depth over total plant dry weight per area, RMF) declined linearly in both years with increasing sowing density (Figure 3A, Table S2) with the exception of cultivar Barke in 2014 which had a constant RMF across sowing densities (see Figure S6). Furthermore, RMF was higher in 2013 than in 2014. In 2013, the plants had not bolted yet, and during stem elongation and bolting relatively much biomass is presumably partitioned to the shoot.

In contrast to RMF, stem mass fraction (SMF) increased linearly in both years with increasing sowing density (Figure 3B, Table S2). Differences in absolute values again reflect the sampling time. In 2014, SMF included ears, which however does not introduce a bias, as density did not influence flowering time. Etiolation is a commonly observed response to competition, however, in our experiments plant height was not significantly affected by sowing density treatment (data not shown) and thus does not explain the increased SMF, rather the increase in SMF is caused by an increase in the number of stems, as reflected by the tiller count. Leaf mass fraction was not affected significantly (Figure 3C, Table S2).

**Figure 4:**
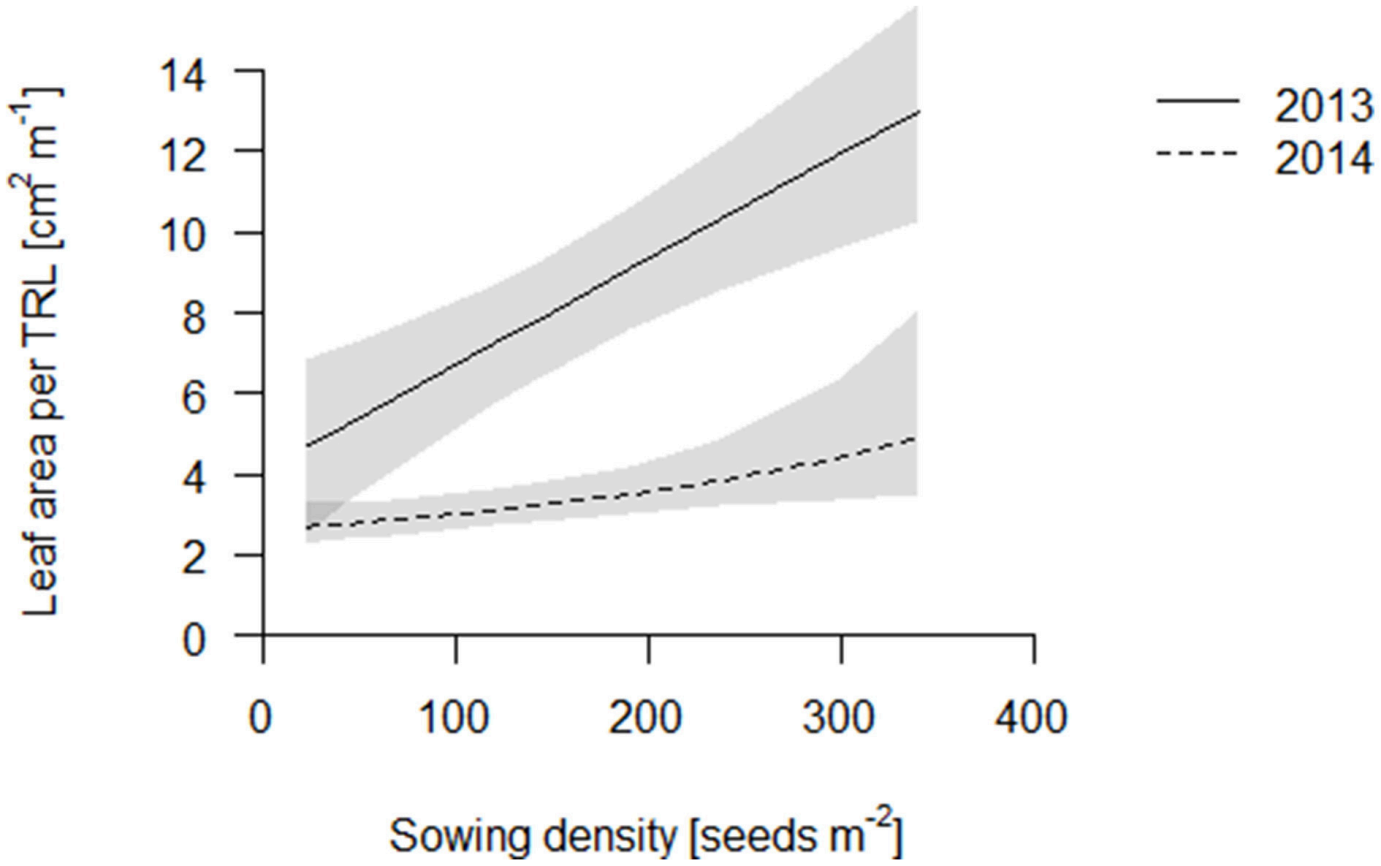
**Leaf area per total root length (TRL) of the coring in 2013 (solid line) and 2014 (dashed line)**. Data are presented as best fits with 95% confidence interval (gray). For equations, *R*^2^ and *p*-values see Table S2.

**Figure 5:**
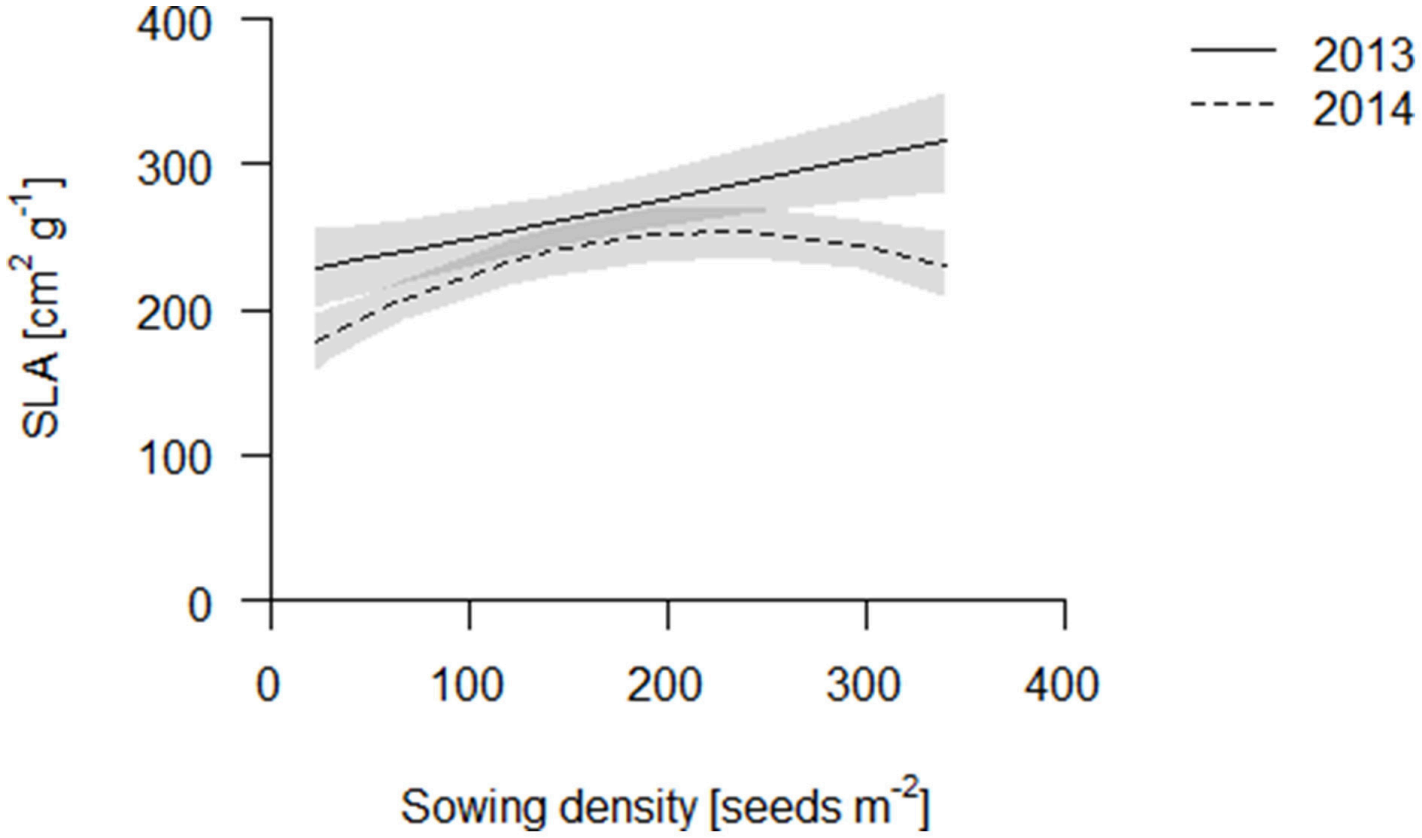
**SLA of the coring in 2013 (solid line) and 2014 (dashed line)**. Data are presented as best fits with 95% confidence interval (gray). For equations, *R*^2^ and *p*-values see Table S2.

We propose that leaf area per total root length may be a better indicator of a functional equilibrium than shoot to root ratios, as carbon fixation (aboveground) and nutrient uptake (belowground) are typically estimated on basis of geometry, not mass. In both years, leaf area per TRL increased with increasing sowing density (Figure 4, Table S2). This increase is partly explained by an increase in specific leaf area (SLA) with increasing density which, given a constant LMF, caused high density plots to have a greater leaf area (Figure 5, Table S2). Hence, plants had thinner but larger area leaves at higher densities. Specific root length (SRL) also increased, but not enough to compensate for the reductions in RMF.

### Sowing density increases SRL in the topsoil

**Figure 6:**
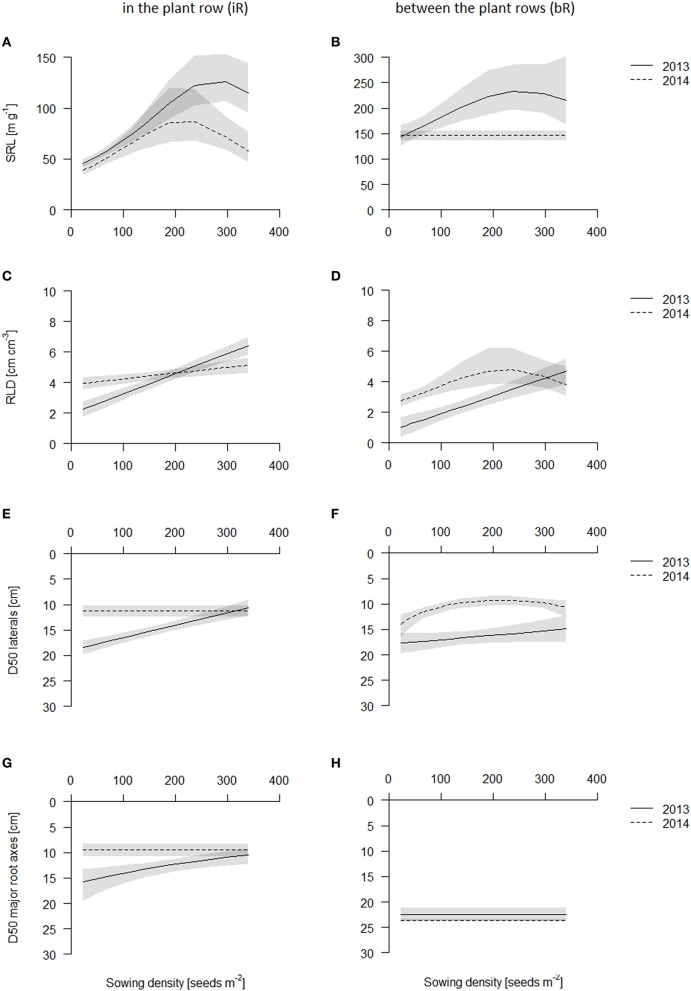
**Belowground traits of the coring in 2013 (solid line) and 2014 (dashed line) in the plant row (iR, left) and between the plant rows (bR, right)**. Data are presented as best fits with 95% confidence interval (gray). For equations, *R*^2^ and *p*-values see Table S3. SRL in the topsoil iR **(A)** and bR **(B)**; RLD in the topsoil iR **(C)** and bR **(D)**; D50 values of laterals iR **(E)** and bR **(F)**; D50 values of major root axes iR **(G)** and bR **(H)**.

Although in 2014 the average SRL was lower than in 2013, trends in both years were the same: sowing density increased SRL strongest in the topsoil layer (0–10 cm) (Figures 6A,B, Table S3, see also Figures S7, S8). SRL increased in the plant row in the topsoil layer (0-10 cm) with increasing sowing density (from 50 cm g^−1^ in 2013 and 40 cm g^−1^ in 2014 to 150 cm g^−1^ in 2013 and 70–80 cm g^−1^ in 2014, Figures S7, S8). SRL in the row, though, was much smaller in the topsoil than in all other soil layer (SRL in layers below 10 cm depth were between 100–250 cm g^−1^ in 2013 and 70–170 cm g^−1^ in 2014), supposedly because the root crowns, where all the nodal roots come together, are in those samples. SRL was in the row highest in 10-20 cm layer (~150 cm g^−1^ in 2013 and 100–110 cm g^−1^ in 2014) and declined slightly with increasing depth (100–150 cm g^−1^ in 2013 and 90–100 cm g^−1^ in 2014) but was at these depths not affected by sowing density (see Figures S7, S8). In contrast to the in the row cores, between the plant rows cores had greatest SRL values in the topsoil (140–150 cm g^−1^ in 2014) and decreased with increasing depth. However, while in 2013 SRL increased with increasing sowing density in the topsoil, in 2014, SRL only increased in the lower sowing densities and was rather constant over sowing density from medium sowing densities on (Figure 6A). In the deeper layers, from 10 cm on, SRL was not affected by sowing density.

### Root length density (RLD) in the topsoil increases with increasing sowing density

In both years, root length density (RLD, cm root per cm^3^ soil) was always highest in the plant row in the topsoil (0–10 cm), where the root crowns are (Figure 6C, see also Figures S9, S10). RLD increased in the row linearly with sowing density in 2013 from 2.5 cm cm^−3^, to 4.5 cm cm^−3^, to 6 cm cm^−3^ for the lowest, medium and highest sowing density, respectively (Figure 6C, Table S3). This increase in RLD was about 2–3-fold. In 2014, at the later harvest date, RLD however did not vary significantly among density treatments and were comparable to the RLD at the higher sowing densities in 2013 (Figure 6C). Between the plant rows, RLD was greatest in the top (0–10 cm) soil and declined with depth with the exception for the low densities in 2013, which had greater RLD deeper down (10–20 or 20–30 cm) (Figure 6D, Figures S9, S10). As for the in the row cores, RLD between the rows in the topsoil increased with increasing sowing density linearly in 2013 from about 1 cm cm^−3^ at the lowest to 3 cm cm^−3^ at the medium and 5 cm cm^−3^ at the highest sowing density (Table S3). In 2014, this effect was less strong and saturated above 200 plants m^−2^ (Figure 6D, Table S3).

### Higher densities had more shallow roots through increases in fine root production in the topsoil

We estimated D50 values (depth of 50% of total root length in the top 40 cm of the core) in order to understand relative root placement. We calculated D50 values for thicker roots with diameters larger than 0.4 mm and thinner roots (diameters of 0.1–0.39 mm) separately, in order to approximately separate major axis from lateral roots. D50 values were always below 20 cm, indicating that plants always placed more roots in the topsoil, except for the thicker roots in between the row, which is supposedly explained by the downward angles at which these major axes grow (Figures 6E,F). The D50 values for the major axis were not influenced by sowing density, while the D50 of the fine roots tended to decrease, i.e., relatively more fine roots were placed in the topsoil, linearly from about 18 cm in lowest sowing density to about 10 cm in highest sowing density. Effects however were noisy, and only significant in 2013 in the row and in 2014 in between the row coring positions (Table S3, see also Figures S11, S12). In 2013, D50 of fine roots decreased in the row with increasing sowing density linearly from about 18 cm in lowest sowing density to about 10 cm in highest sowing density (Figure 6E).

## Discussion

We investigated how biomass partitioning to roots, root length density (RLD) and root distribution are influenced by sowing density by establishing dose-response curves to a wide range of sowing densities in the field. Earliest effects of sowing density on individual plants were visible around 30 DAS in the shoot and increased thereafter. Around anthesis (flowering), sowing density had strong effects on individual plants, affecting size, biomass partitioning and morphology of both roots and shoot. At the field level, canopy closure, leaf area index (data not shown), total shoot biomass, total root biomass, biomass partitioning, and relative rooting depth (as D50) were affected. Eventually, yield maximized at about 230 plants m^−2^, which means that increased biomass (g ha^−1^) and RLD at 340 plants m^−2^ did not translate into further yield increases, rather harvest index declined. Our results may have consequences for extrapolating root function from the individual plant level to the field level and thus require careful (re)evaluation of the utility of root traits in the field, and the interpretation of genotypic contrasts observed in greenhouse-based phenotyping platforms, especially, since in greenhouse studies usually single plants are investigated.

### Root mass fraction decreases with sowing density, while stem mass fraction increases

Root mass fraction (RMF) of non-woody species typically reduces over time as plants grow larger (Poorter and Sack, 2012; Wang et al., 2015; Yin and Schapendonk, 2004; and specifically for barley Kamel, 1959). Ontogenetically, we may thus expect RMF to increase at high plant densities, as plants are smaller in high densities and smaller plants have greater RMF. So far, a variety of outcomes have been found for RMF response to plant density: Berendse and Möller (2009), for example, found such increased RMF with increasing density for *Plantago lanceolata* under low N supply, but not under high N supply, and concluded that increased RMF to plant density was better explained by plasticity responses to reduced nutrient availability in the denser populations. Under high/normal nutrient availability, Kamel (1959) did not find effects of plant density on the shoot to root ratios in barley, suggesting that plants exhibited neither plastic responses to reduced nutrient availability, nor ontogentic drift. Ågren and Franklin (2003), however, found shoot mass fraction to increase with increasing plant N concentration, suggesting that RMF actually declines at very high fertilization. Our results, also obtained under non-limiting fertilization levels, show a decrease in RMF in response to plant density and may be interpreted as an adaptive response to light competition.

Based on meta-analysis, Poorter et al. (2012) found that on average species tended to increase their SMF and specific stem length in response to density. Increased SMF and specific stem length are a possible reaction to competition for light assuming that plants elongate and increase their height in order to avoid shading by neighboring plants. Such etiolation responses have been nicely demonstrated by Nagashima and Hikosaka (2011) who placed pots with *Chenopodium album* at different heights and observed that the lowered plants simply stretched more such that they reached the same height as the higher plants. Similar to Poorter et al. (2012), we observed an increase in SMF. This increase in SMF did not correlate with an increase in crop height at anthesis, although stem elongation started slightly earlier in 2013 in the high densities (data not shown) giving rise to small differences in plant height during this earlier stage. Reports in the literature on plant height vary, with some finding increases (Munir, 2002), while others finding decreases (Turk et al., 2003; Soleymani et al., 2011) and yet others both increases and at very high densities decreases in plant height with increasing sowing density (Farnia et al., 2014). Conflicting reports may be caused by the fact that high competition for light might trigger an etiolation response, but that at the same time height is tempered for allometric reasons or reduced nutrient availability.

Crop species might also have lost the etiolation response as breeders have purposely targeted short straw varieties in order to reduce lodging risk and increase harvest index. Kiaer et al. (2013) concluded from a meta-analysis that crops are generally less competitive than wild species and that this is the result of selection under high-resource availability and weed-free conditions in which competitive ability was less important. If so, we may expect that crop height does not respond to sowing densities, which is what we observed in our two barley cultivars. The increase in SMF is thus not due to increased height but rather smaller plants had more tillers relative to their size, and thus more stems (about 80% of tillers carried ears, except in the three lowest sowing densities, where only 60% of the tillers carried ears, data not shown) and consequently the stem density per area was greatest at the highest sowing densities.

### The highest densities had the greatest tiller density and biomass production

The tiller count for the highest sowing densities (>140 seeds m^−2^) reached a maximum tiller count of 2–5 tillers per plant in both years at about 5 weeks after sowing, only 1 week after the earliest response of tiller counts to sowing density. Tillering in *Poaceae* is known to respond early to density, not due to direct competition but to changes in red to far-red ratios (Casal, 2013). Despite the early arrest in tillering, the highest sowing densities reached the highest tiller density of 2000 tillers per m^2^ (1500 tillers per m^2^ in 2013), which is two to three times greater than found in other studies (Finlay et al., 1971; Fukai et al., 1990; Munir, 2002; Soleymani et al., 2011). However, this difference probably reflects the contrast between the temperate climate of Germany and the arid climates of Jordan, Iran, and Australia. In Germany, guidelines recommend a spike density of 800–1150 spikes per m^2^ (Landwirtschaftskammer Nordrhein-Westfalen) and with only 80% of the tillers carrying a spike we achieved 1000–1400 spikes per m^2^. Surprisingly, this high tiller density did not compromise the dry weight per tiller in our study, such that not only the tiller density but also the shoot biomass per ha increased linearly with density which stand in contrast with the constant final yield concept (Weiner and Freckleton, 2010). Possibly constant yield is achieved later in time as observed by Fukai et al. (1990). Final constant yield does not usually distinguish between shoots and grain biomass. In our study, we found something similar to constant final yield in terms of grain yield, but not in terms of shoot biomass.

Grain yield was slightly reduced at the highest density, but even if grain yield was constant, the presumptive harvest index (final grain yield over total biomass at final harvest; in our study, we can only approximate harvest index by using biomass data from the coring events; data not shown) clearly declined with increasing density (assuming that stem and leaf mass did not increase drastically after anthesis). This decline in harvest index was also observed by Farnia et al. (2014) and may suggest that the higher biomass production was metabolically costly and reduced yield.

### Root length density increases with sowing density due to greater specific root length

The net effect of increasing total biomass but decreasing biomass partitioning to roots is that the total root biomass per ha stayed constant, or increased possibly slightly, but in general the plot to plot variation was large (data not shown). Thus, the increase in RLD in the topsoil is mostly caused by an increase in SRL, since root biomass itself stayed constant. Aerts (1999) lists several studies in which plants respond to interspecific competition by reducing RMF and increasing SRL and we thus may have observed a common response. Changes in SRL are, however, difficult to interpret as they may be caused by shifts in root anatomy, or in the relative production of fine roots verses major axis roots. In recent years, more attention has been drawn to root anatomical traits and their function (reviewed by Paez-Garcia et al., 2015), and for barley root cortical senescence may be of special importance (Schneider et al., in revision).

Determining changes in root anatomy of cored roots seems difficult, as we do not know the age or class of the root fragments that we collected and thereby have no indication if root anatomy, and in particular the rate of cortical senescence, was affected by sowing density.

Our data suggests that increases in SRL at least in part were caused by a greater portion of fine roots in the topsoil. Several studies have suggested that interspecific competition may lead to increased root proliferation (Mommer et al., 2010), and thereby increased production of lateral roots. Although these responses are not completely understood, one explanation may be that plants try to outcompete by depleting soil resources faster than their neighbors. Such responses would not be desirable in agriculture, as it would not increase the performance of the whole crop. Rather the crop should exhibit lateral root traits that maximize resource acquisition, as for example recently estimated using a root model by Postma et al. (2014a) who showed that high rooting density may increase phosphorus acquisition, but not nitrate acquisition unless nitrate concentrations are very high.

### Greater placement of roots in the topsoil with increasing density

In our study, plants accumulated root length in the topsoil supporting our hypothesis. Topsoil foraging is important for the acquisition of immobile nutrients (Dunbabin et al., 2003), and possibly reduces leaching of mobile nutrients (Thorup-Kristensen, 2001). Many crops and plant species explore the topsoil with greater intensity, for wheat see Schweiger et al. (2009) and Lotfollahi (2010) and for barley Lampurlanés et al. (2001) and Breuning Madsen (1985), who showed that the topsoil is foraged with greater intensity irrespective of soil or tillage type. Topsoil foraging has been strongly associated with changes in rooting angles of the major axis (Lynch, 2013). Our coring data suggests however that the depth of the major root axis did not change over sowing density for the two genotypes. Hence, the difference in D50 for the major axis between in the row and in between the row columns over the half row distance is 8/10 and 14/10 for 2013 and 2014, respectively. We think that these calculations may reflect average angles of arctan(10/8) = 51° and arctan(10/14) = 36° degrees from vertical for both years respectively, although the year difference is somewhat artificially caused by the “in the row” D50 values.

### Changes in biomass allocation and root morphology and architecture may be adaptive responses to plant competition

Increases in plant density cause the resource availability per plant to decline. Mathematically, we might assume that both light availability and nutrient availability scale linearly with the area per plant. Thus reductions in plant size could be simply seen as a decline in resource availability per plant. Plant growth, however, is not always a pure function of resource availability, neither does one resource alone usually determine production (law of the minimum), as plants can adapt their architecture and morphology in order to increase or balance resource capture (Ågren et al., 2012; Dathe et al., 2013; Postma et al., 2014b).

Our data shows that barley plants adapt their biomass allocation, root and shoot morphology, and architecture in response to plant density. Since biomass production increased with density, our results suggest that these adaptations increased resource capture of the whole crop. Shoot:root ratios are thought to express a functional equilibrium between above- and belowground (Gleeson and Tilman, 1992). We computed leaf area per root length as a better expression of that functional equilibrium as it takes changes in SRL and SLA into account. Current models of nutrient uptake and photosynthesis typically integrate over area or length, not mass (Thornley, 1995; Boote et al., 2013; Dunbabin et al., 2013). In both years, leaf area per root length increased with increasing sowing density. This may suggest that increasing sowing density shifted plants into carbon limited growth. Through fertilization, farmers try to achieve yields that are not limited by nutrient capture, and consequently light capture is probably the limiting factor once a crop is established.

At high nutrition, Grime and Hodgson (1987) suggest that ideal competitive traits are fast shoot growth to avoid shading by neighbors, high relative growth rates and high morphological plasticity. Our measurements on shoot traits seem to at least partly confirm these traits, however, we also observed changes in the root traits. These suggest that the metabolic efficiency of the root system, that is the relative investment of biomass (carbon, N, P) into roots, is reduced while the nutrient uptake capacity of the root system is increased by increasing root length. These increases occurred mainly in the topsoil.

Simulation studies suggest that under agricultural conditions root competition for immobile nutrients is relatively low (Postma and Lynch, 2012; Postma et al., 2014a) and thus further increases in RLD probably do translate into greater uptake of nutrients in the topsoil. Acquisition of nitrogen may be improved through increased NH_4_ uptake although NH_4_ are typically low in well-aerated temperate soils. Effective nitrate uptake is thought to be associated with low RLD and exploration of large soil volumes, which is in the case of crops translates into deep rooting (Dathe et al., in press). We did not find differences in RLD deeper down, however, such that we cannot exclude that density may influence RLD below 60 cm. We conclude that at high sowing density, fertilized barley produces more leaf area through increased SLA, more stem biomass through increased allocation to stems, and more root length in the topsoil through increased SRL. Maximum yield was found around 230 plants m^−2^, as in higher densities the harvest index declined, possibly due to over commitment to shoot biomass, while total light capture at the crop level was not increased.

### The strong effects of sowing density on individual plant traits raises questions about how to scale up research results from the lab to the field

Currently, most root research is performed on individual plants growing in pots, relatively isolated from other plants. However, if root research is to have an impact on breeding strategies and agriculture as a whole (Kuijken et al., 2015), we need to understand how roots function in the context of high plant densities. Our research shows that high plant density can drastically change the root system, the relative rooting depth (D50), the biomass partitioning to roots and the root length distribution with depth. Changes in biomass partitioning and root morphology or architecture supposedly influence the functioning of the root system (Berendse and Möller, 2009; Lynch, 2013), and may partly compensate for increased competition at higher density, thus increasing biomass production and yield of the whole crop. In our research, both cultivars were very similar in nearly all aspects and responded similarly to sowing density. We suspect, however, that genotypic differences in response to plant density exist, since barley genotypes can differ quite dramatically in their responses to other factors like e.g., nutrient availability (Ayad et al., 2010; Karley et al., 2011). Plant performance of different genotypes and the utility of traits are often evaluated on the basis of early vigorous growth in plants growing at low densities. However, these genotypes may well lose their advantage in a high density stand. The highest yield in this study was obtained with relatively small individual plants, a high harvest index, and reduced biomass allocation belowground, while total crop nutrient uptake was guaranteed by high RLD and greater SRL, as, for example, maize showed to increase RLD under low N especially in the topsoil (Mu et al., 2015) and wheat plants with more roots (unpruned plants) had a greater N uptake under competition than plants with fewer roots (pruned plants) (Andrews and Newman (1970). Furthermore, early biomass production may simply result in early competition (Weiner and Freckleton, 2010), and not translate into yield. We suggest that variation in individual plant size during early growth stages may be compensated for by variation in plant density and thus has little meaning for agricultural production. We conclude that genotypes should not just be evaluated on absolute values, but rather in terms of efficiencies, such as root metabolic efficiency, harvest index, and nutrient uptake efficiency (uptake per unit root mass) as these characteristics are more important at the crop level than individual plant weight, which can be compensated for by sowing density.

## Conclusions

Sowing density influenced individual plant size and relative biomass allocation to different plant organs. The changes in biomass allocation are opposite from what we may expect from general allometric rules; that is bigger plants have reduced RMF and increased SMF and LMF. Thereby, the changes in biomass allocation are not just related to size, but related to (adaptive) responses to competition. Our results indicate that plant density increased the SMF at the cost of the RMF. Increased SRL and increased overall biomass production allowed the high sowing density plants to maintain relatively high RLDs in soil, despite the reduced biomass allocation to roots. Sowing density increased RLD in the topsoil, especially in between the row, while not affecting the RLD further down. This may mean that deep rooting, at least in rather light- than nutrient-limited systems, is not sensitive to sowing density, as we initially hypothesized.

Plants reduced the investment into root biomass with increasing sowing densities and simultaneously enhanced the investment of this root biomass into fine roots. Moreover, aboveground, biomass was invested into stems; however, although, plants did not raise the investment into leaf biomass fractions with increasing sowing density, they increased leaf area, by increasing SLA. The combination of these changes in allocation indicates that the plants in our study were generally more aboveground light-limited than belowground resource-limited.

Changes in root length distribution with depth, SRL and overall biomass allocation to roots suggest that architectural and morphological changes in the root system occurred, and possibly greater source limited tradeoffs for root growth had taken place. While these hypotheses require further investigation, they possibly have important consequences for root phenotyping of isolated plants, the functional interpretation of traits for nutrient and water acquisition, and the importance of traits that may increase the metabolic efficiency of the root system.

## Author contributions

The conception and design of the work was performed by all authors. VH and JP acquired the data. VH processed the samples. Data analysis was done by VH and JP. Data interpretation was done by all authors. VH performed the drafting of the work with accompanying input from JP. All authors revised the manuscript critically for important intellectual content and approved the final version of the manuscript. Further, all authors agreed to be accountable for all aspects of the work.

### Conflict of interest statement

The authors declare that the research was conducted in the absence of any commercial or financial relationships that could be construed as a potential conflict of interest.

## Footnotes

^1^http://www.dlr.rlp.de/Internet/global/inetcntr.nsf/dlr_web_full.xsp?src=7647GJT68H&p1=V3802SO2OW&p2=M42BIW88I8&p3=9203R4M5VS (last accessed: January 12, 2016); http://www.am.rlp.de/Internet/AM/NotesAM.nsf/amweb/6d6fa012f043c619c1257171002e8a75?OpenDocument&TableRow=2.7 (last accessed: January 12, 2016).

^2^Richtlinien für die Durchführung von landwirtschaflichen Wertprüfungen und Sortenversuchen (2014). 2.8.1–2.8.29.

## Abbreviations

BBCH: crop developmental stages encoded in decimal numbers developed by BASF, Bayer, Ciba-Geigy and Hoechst after (Lancashire et al., 1991)
D50: median of the interpolated root length distribution, i.e., the depth (in cm) which divides the root length in the core into equal parts (50% of root length above and 50% below that D50 value), calculated on root length up to 40 cm depth
DAS: days after sowing
LMF: leaf mass fraction
PAR: photosynthetic active radiation
RDW: root dry weight
RLD: root length density
RMF: root mass fraction
SLA: specific leaf area
SMF: stem mass fraction
SRL: specific root length
TPDW: total plant dry weight.
