# Supplementary material for: Sowing Density: A Neglected Factor Fundamentally Affecting Root Distribution and Biomass Allocation of Field Grown Spring Barley (Hordeum Vulgare L.)
Source: Front Plant Sci. 2016 Jun 28;7:944. doi: 10.3389/fpls.2016.00944 (PMC4923255; doi:10.3389/fpls.2016.00944)
Supplement: Supplementary file 1 [file DataSheet1.pdf]

Supplemental Material

Sowing Density: A Neglected Factor Fundamentally Affecting Root Distribution and Biomass Allocation of Field Grown Spring Barley (Hordeum Vulgare L.)

Hecht, V.L., Temperton, V.M., Nagel, K.A., Rascher, U., Postma, J.A.
*** Correspondence: Corresponding Author: j.postma@fz-juelich.de**

1. Supplementary Tables

Table S1 | Best fits of the three different applied models for shoot traits. Only coefficients with p<0.05 were taken into account.

**Trait**	**Year/Best fit**	**a**	**b**	**c**	**Adjusted R^2**	**Multiple R^2 / *Residual Standard error***	**p-value**	**Degrees of freedom**
Tillers per area	2013/model 1	365.2617	4.6873		0.5672	0.5751	1.29E-11	54
	2014/model 1	696.6742	2.1879		0.6362	0.6492	1.34E-07	27
Tillers per plant	2013/model 2	0.04475	0.0008944	-1.296E-06	0.5044	0.5225	3.12E-09	53
	2014/model 2	0.006835	0.001136	-1.271E-06	0.8714	0.8806	1.00E-12	26
Shoot dry weight per area	2013/model 1	114.8576	1.9358		0.6152	0.6222	5.24E-13	54
	2014/model 1	1055.9095	2.7742		0.3311	0.355	0.0006495	27
Final grain yield	2013/model 3	-0.7774	8.4607	26.9934		*0.5949*		97
	2014/model 2	0.2013	-0.0005279	1.111E-06	0.6017	0.6301	2.42E-0.6	26
Shoot dry weight per tiller	2013	0.3396						
	2014	1.39987						

Table S2 | Best fits of the three tested models of plants ratios in 2013 and 2014. Only coefficients with $p<0.05$ are taken into account.

**Trait**	**Year/Best fit**	**a**	**b**	**c**	**Adjusted R^2**	**Multiple R^2**	**p-value**	**Degrees of freedom**
RMF	2013/model 1	0.2964	-0.000581		0.4882	0.501	2.272e-07	39
	2014/model 1	0.08522	-0.0001027		0.2984	0.3235	0.001285	27
SMF	2013/model 1	0.3108786	0.0004072		0.1641	0.185	0.005004	39
	2014/model 1	0.8113	1.354e-04		0.1657	0.1955	0.01633	27
LMF	2013	0.3987						
	2014	0.09849						
Leaf area per TRL	2013/model 1	4.086772	0.026110		0.3122	0.3299	0.0001065	38
	2014/model2	0.3838585	0.0005243		0.2072	0.2355	0.007619	27
SLA	2013/model 1	221.79021	0.27537		0.1766	0.1921	0.0009171	52
	2014/model 1	159.7	8.228e-01	-1.799e-03	0.4764	0.5138	8.482e-05	26

Table S3 | Best fits of the three tested models for root traits of Scarlett and Barke in 2013 and 2014. Only coefficients with p<0.05 were taken into account.

**Trait**	**Year/Best fit**	**a**	**b**	**c**	**Adjusted R^2**	**Multiple R^2**	**p-value**	**Degrees of freedom**
SRL iR 0-10 cm	2013/model 2	0.0246	-1.21E-04	2.18E-07	0.765	0.7765	2.05E-13	39
	2014/model 2	0.0293	-1.65E-04	3.81E-07	0.4987	0.5345	4.82E-05	26
SRL bR 0-10 cm	2013/model 2	0.00754	-2.55E-05	4.99E-08	0.4519	0.5096	0.002341	17
	2014	146.4546102						
RLD iR 0-10 cm	2013/model 1	1.93841	0.01316		0.6897	0.6973	6.207E-12	40
	2014/model 1	3.856821	0.003760		0.2728	0.2988	0.002153	27
RLD bR 0-10 cm	2013/model 1	0.748647	0.01157		0.6659	0.6834	7.006E-06	18
	2014/model 2	0.4057	-1.78E-03	3.99E-06	0.3269	0.375	0.00222	26
D50 iR laterals	2013/model 1	18.990279	-0.0246		0.5276	0.5391	3.13E-08	40
	2014	11.2954127						
D50 bR laterals	2013/model 1	17.913677	-0.008759 *n.s.*		0.06646	0.1156	0.1425	18
	2014/model 2	0.06193	4.28E-04	-9.85E-07	0.3759	0.4205	0.0008309	26
D50 iR major axes	2013/model 2	0.06082	1.024e-04		0.1861	0.206	0.002538	40
	2014	9.531474927						
D50 bR major axes	2013	22.52591285						
	2014	23.57903486						

2. Supplementary Figures

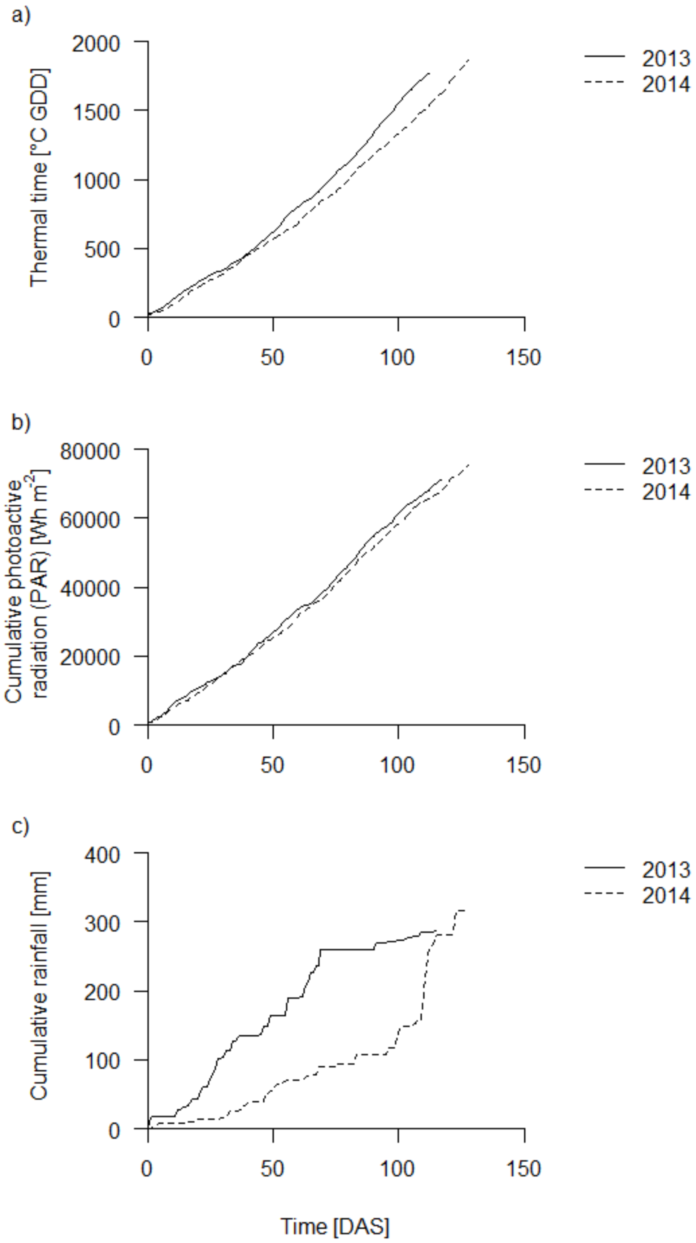

Figure S1 | Climate data for Klein-Altendorf field site in 2013 (solid line) and 2014 (dashed line) during the growing season over time in days after sowing (DAS). a) Thermal time in growing degree days (GDD, average of daily maximum and minimum temperature minus base temperature (here, base temperature = 0°C), adapted according to McMaster and Wilhelm (1997)); b) cumulative incoming photosynthetically active radiation (PAR); c) cumulative rainfall. Data are available under http://www.am.rlp.de/Internet/AM/NotesAM.nsf/amweb/6d6fa012f043c619c1257171002e8a75?OpenDocument&TableRow=2.7.

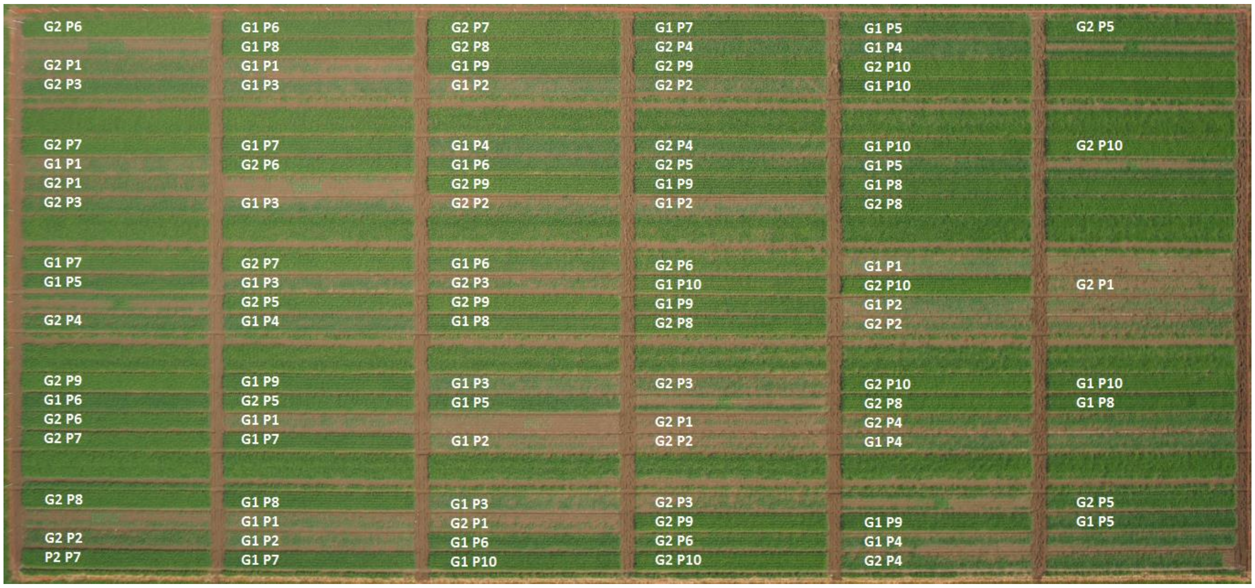

Figure S2 | Experimental design of 2013 at 47 DAS (572.482 °C GDD (growing degree days=average of daily maximum and minimum temperature minus base temperature (here, base temperature = 0°C), adapted according to McMaster and Wilhelm (1997)). G1 and G2 refer to cultivar Scarlett and Barke. P1 to P10 stand for the 10 different sowing densities: 24, 31, 43, 68, 120, 140, 190, 238, 298, and 340 seeds m^{-2}, respectively. Plots were 14.2 m long and 1.5 m wide. Data of the plots not used within this publication are left blank. Note that canopy closure had not yet happened in low sowing densities but from P5 (=120 seeds m^{-2}) on only. Photo with permission of A. Burkart (2013).

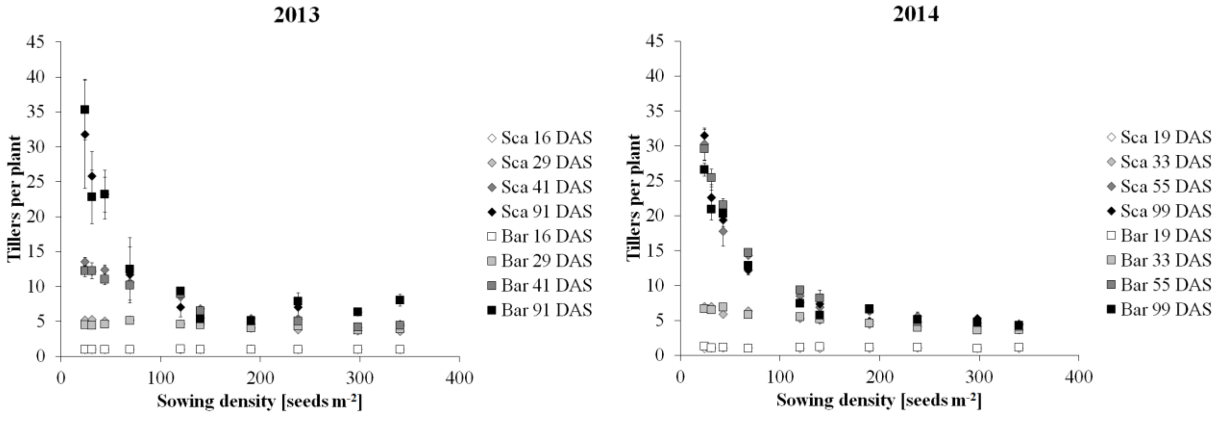

Figure S3 | Tillers per plant counted over the season in 2013 (left) and 2014 (right) for the two genotypes Scarlett (Sca) and Barke (Bar). Values are means (n=5), error bars indicate SEM.

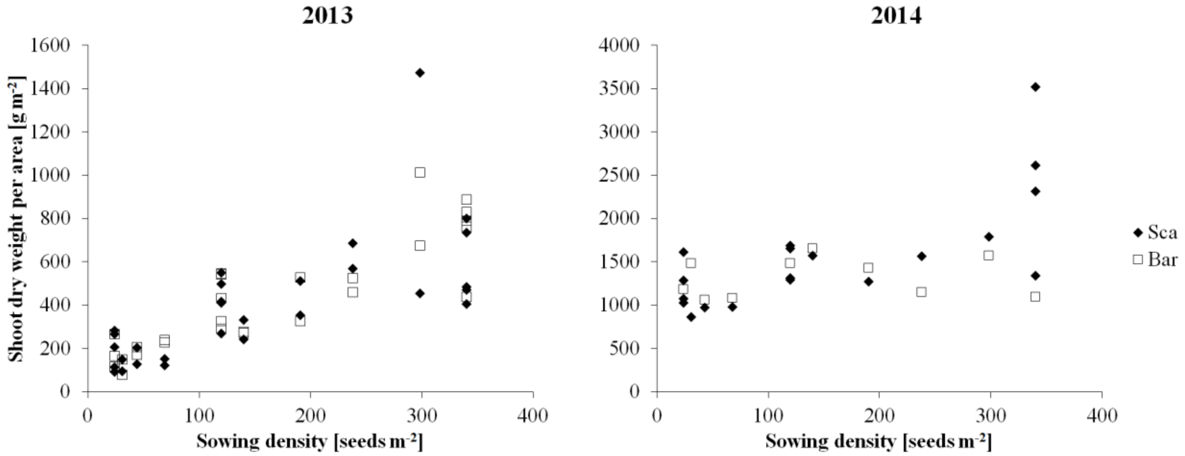

1

Figure S4 | Shoot dry weight per area at the coring event in 2013 (left) and 2014 (right) for Scarlett (Sca) and Barke (Bar). Data are raw data of shoot dry weight per plant multiplied by the corresponding sowing density. Note the different y-axis.

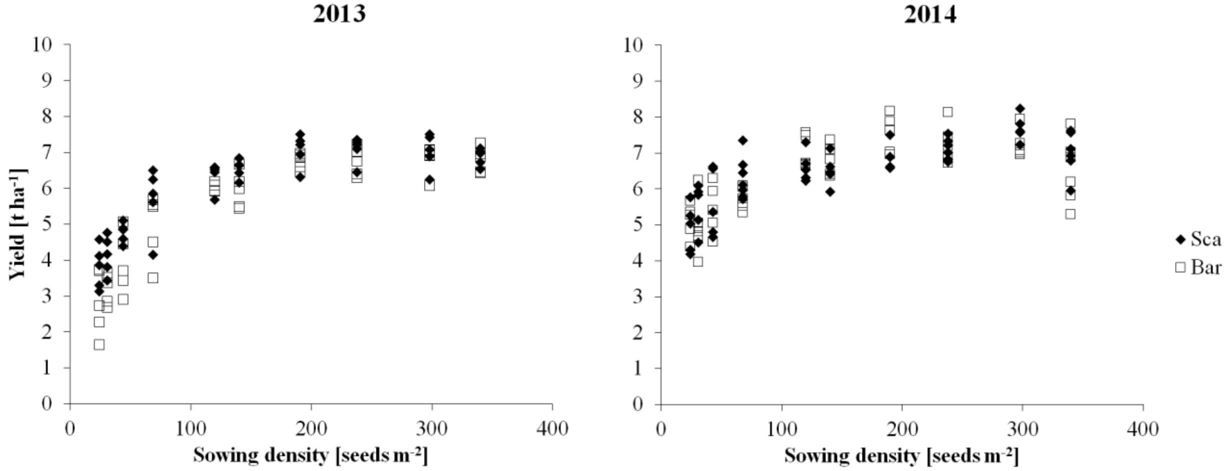

2

Figure S5 | Final grain yield per area in 2013 (left) and 2014 (right) for Scarlett (Sca) and Barke (Bar). Data are raw data.

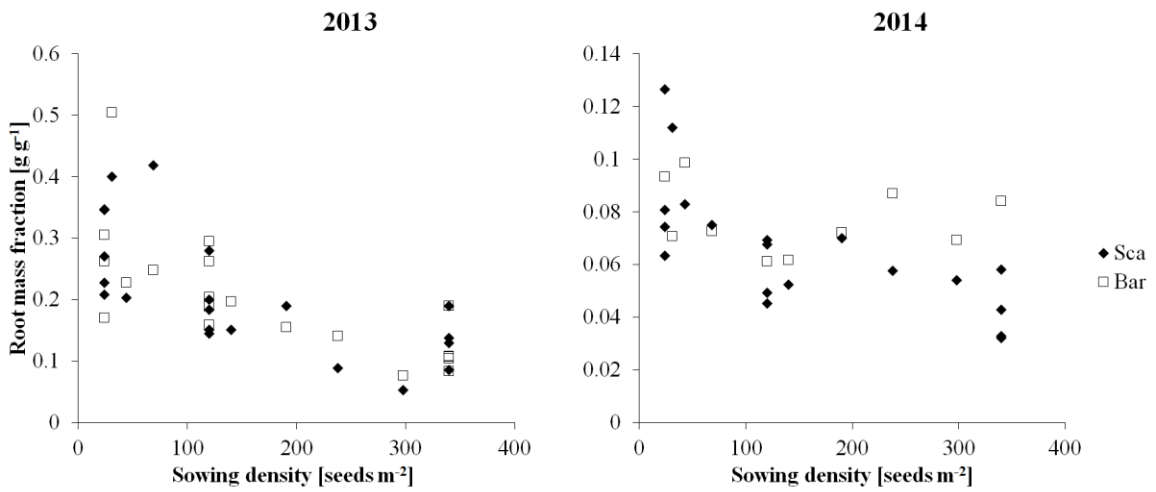

Figure S6 | Root mass fraction (RMF) at the coring event in 2013 (left) and 2014 (right) for Scarlett (Sca) and Barke (Bar). Note the different y-axis. Data are raw data.

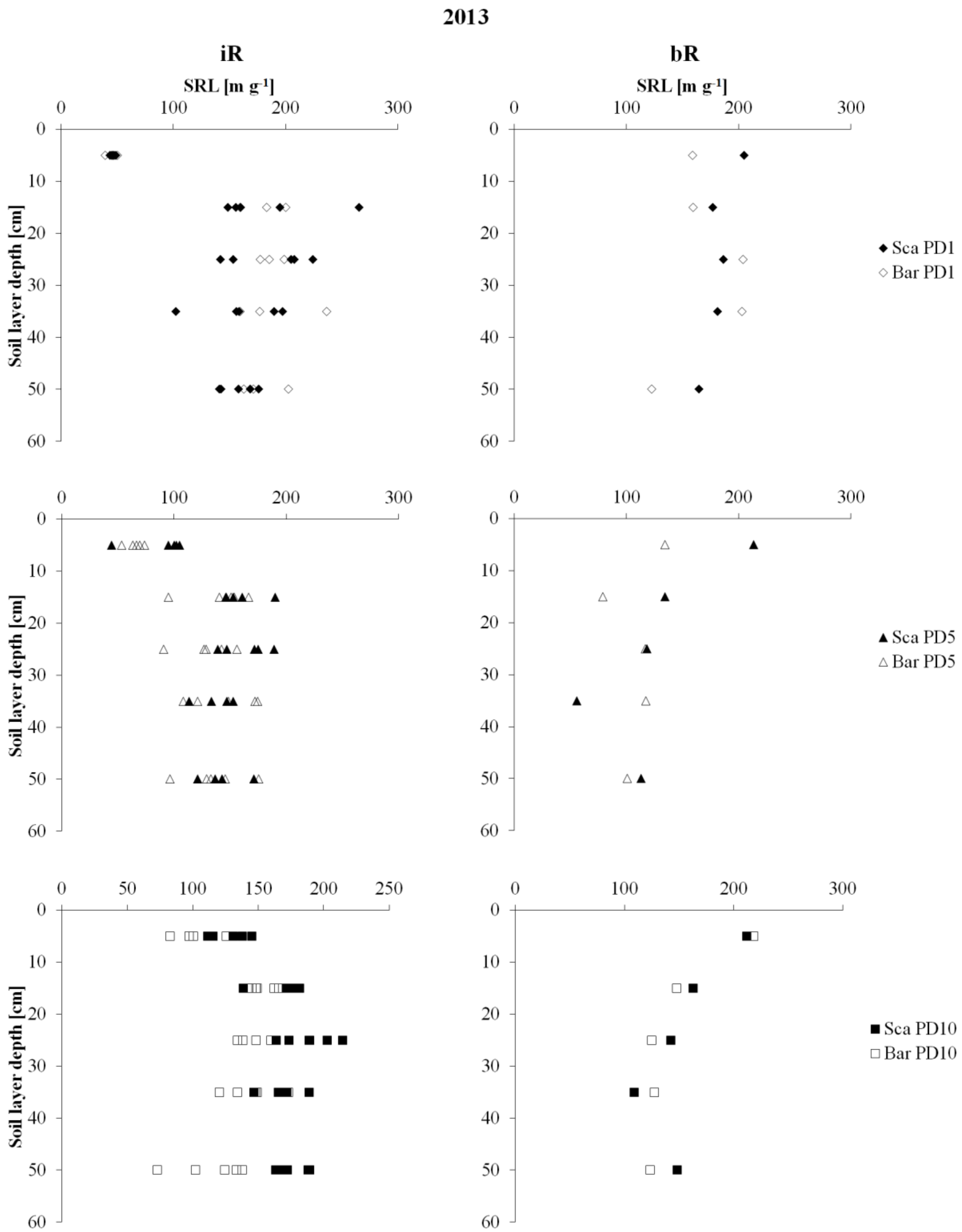

Figure S7 | Specific root length (SRL) in 2013 in the row (iR) (left) and between the rows (bR) (right) for Scarlett (Sca) and Barke (Bar) over the 60 cm depth profile for the lowest (PD1, 24 seeds m^{-2}), medium (PD5, 120 seeds m^{-2}) and highest sowing density (PD10, 340 seeds m^{-2}). Data are raw data.

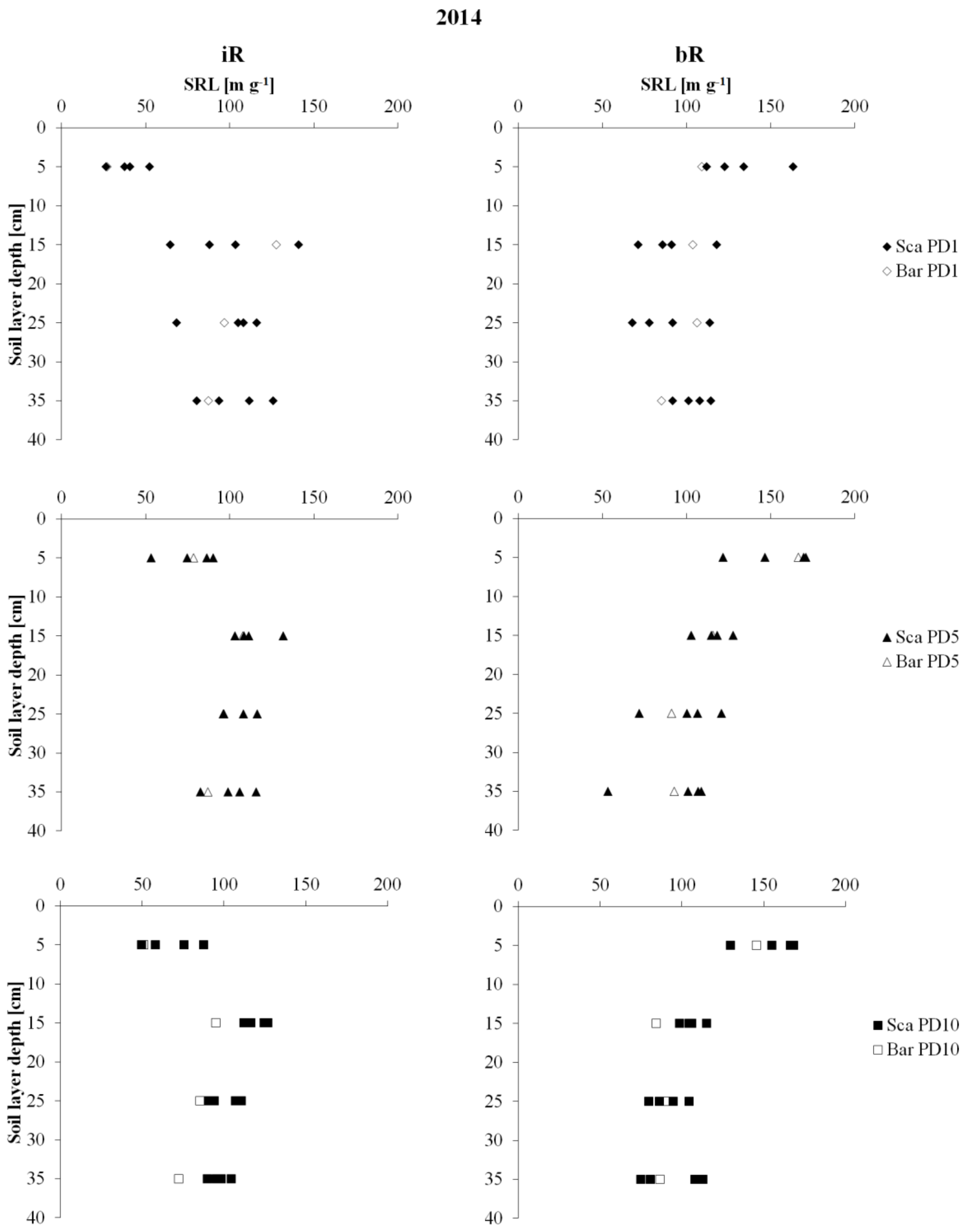

1

Figure S8 | Specific root length (SRL) in 2014 in the row (iR) (left) and between the rows (bR) (right) for Scarlett (Sca) and Barke (Bar) over the 40 cm depth profile for the lowest (PD1, 24 seeds m^{-2}), medium (PD5, 120 seeds m^{-2}) and highest sowing density (PD10, 340 seeds m^{-2}). Data are raw data.

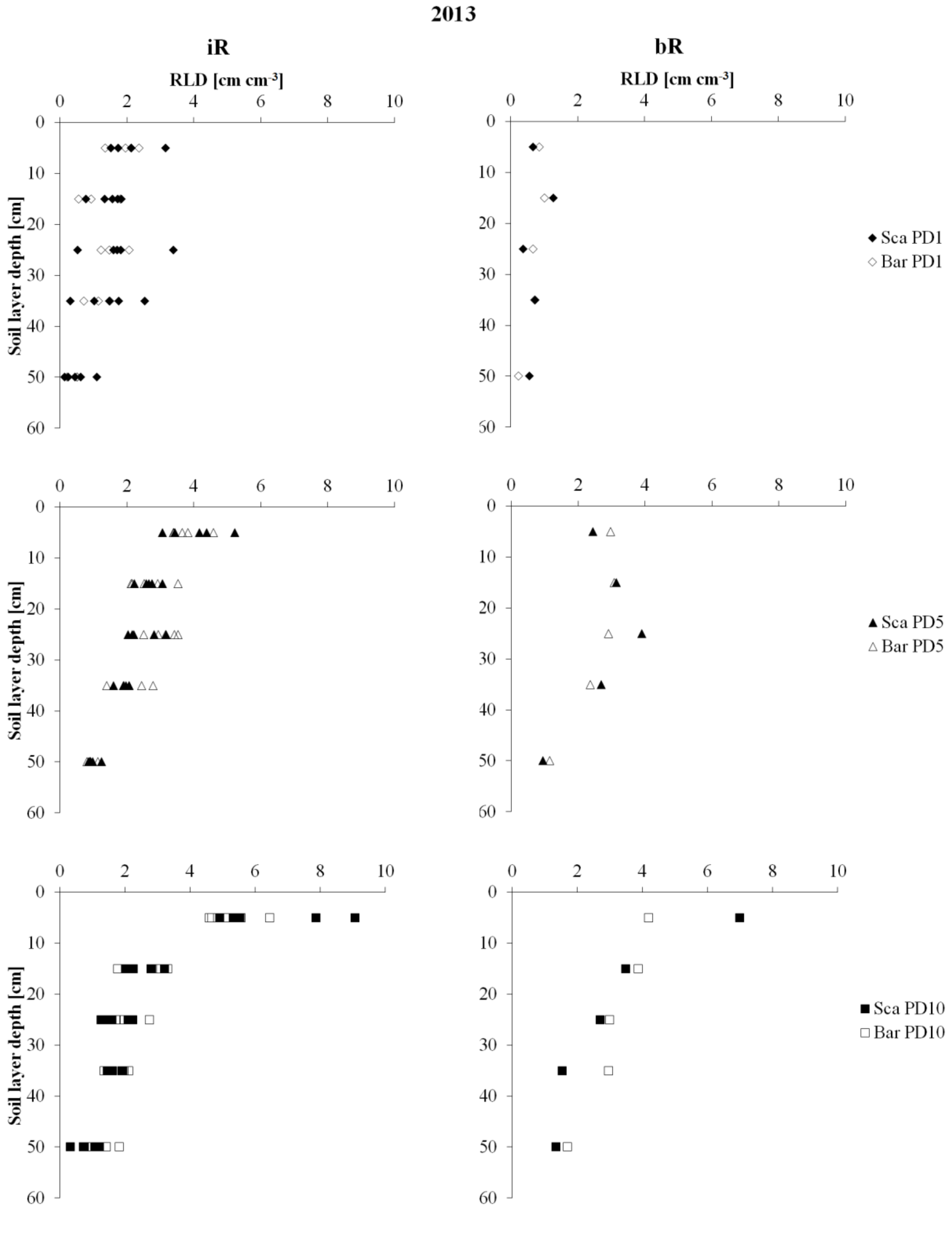

Figure S9 | Root length density (RLD) in 2013 in the row (iR) (left) and between the rows (bR) (right) for Scarlett (Sca) and Barke (Bar) over the 60 cm depth profile for the lowest (PD1, 24 seeds m^{-2}), medium (PD5, 120 seeds m^{-2}) and highest sowing density (PD10, 340 seeds m^{-2}). Data are raw data.

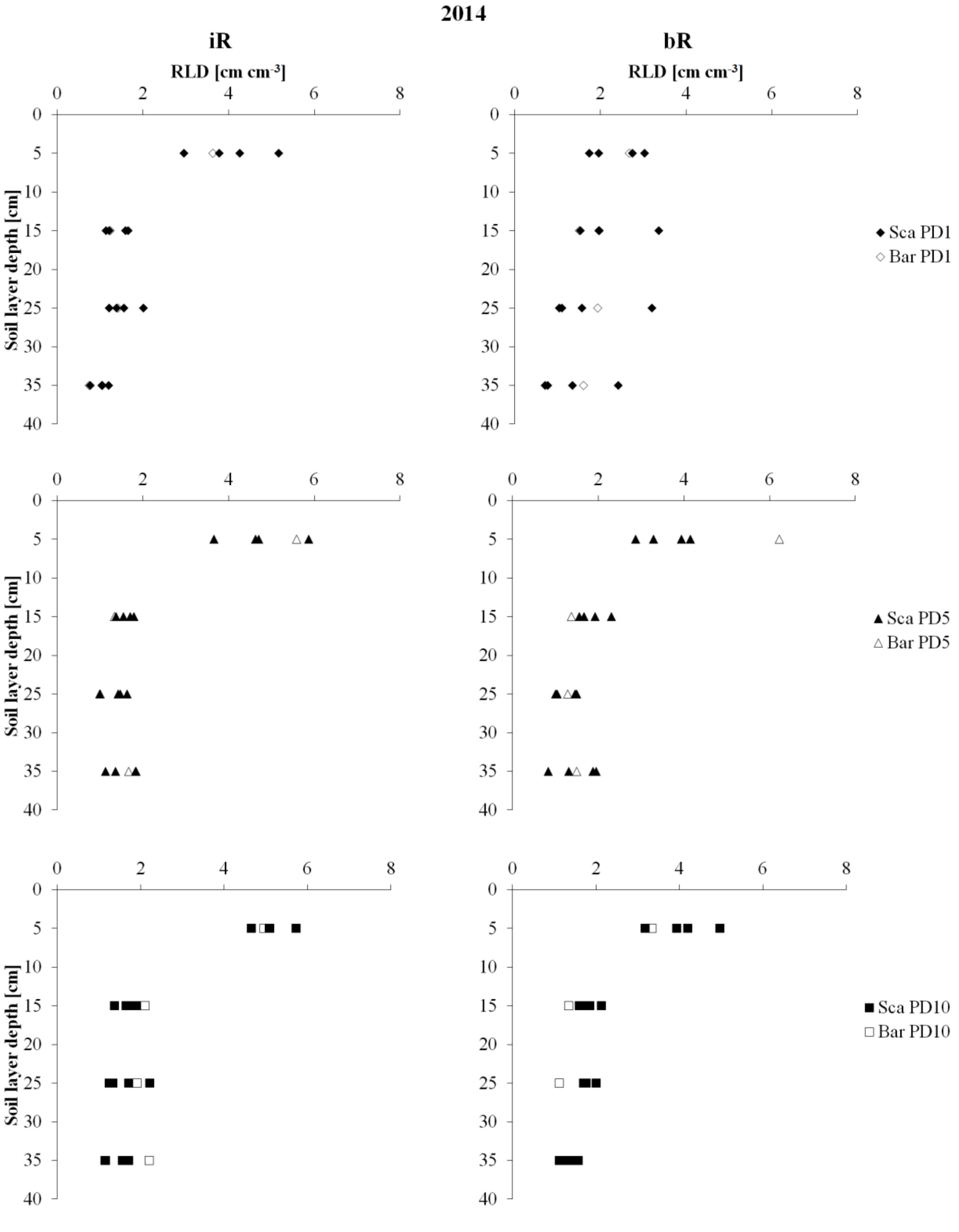

Figure S10 | Root length density (RLD) in 2014 in the row (iR) (left) and between the rows (bR) (right) for Scarlett (Sca) and Barke (Bar) over the 40 cm depth profile for the lowest (PD1, 24

seeds m^{-2}), medium (PD5, 120 seeds m^{-2}) and highest sowing density (PD10, 340 seeds m^{-2}). Data are raw data.

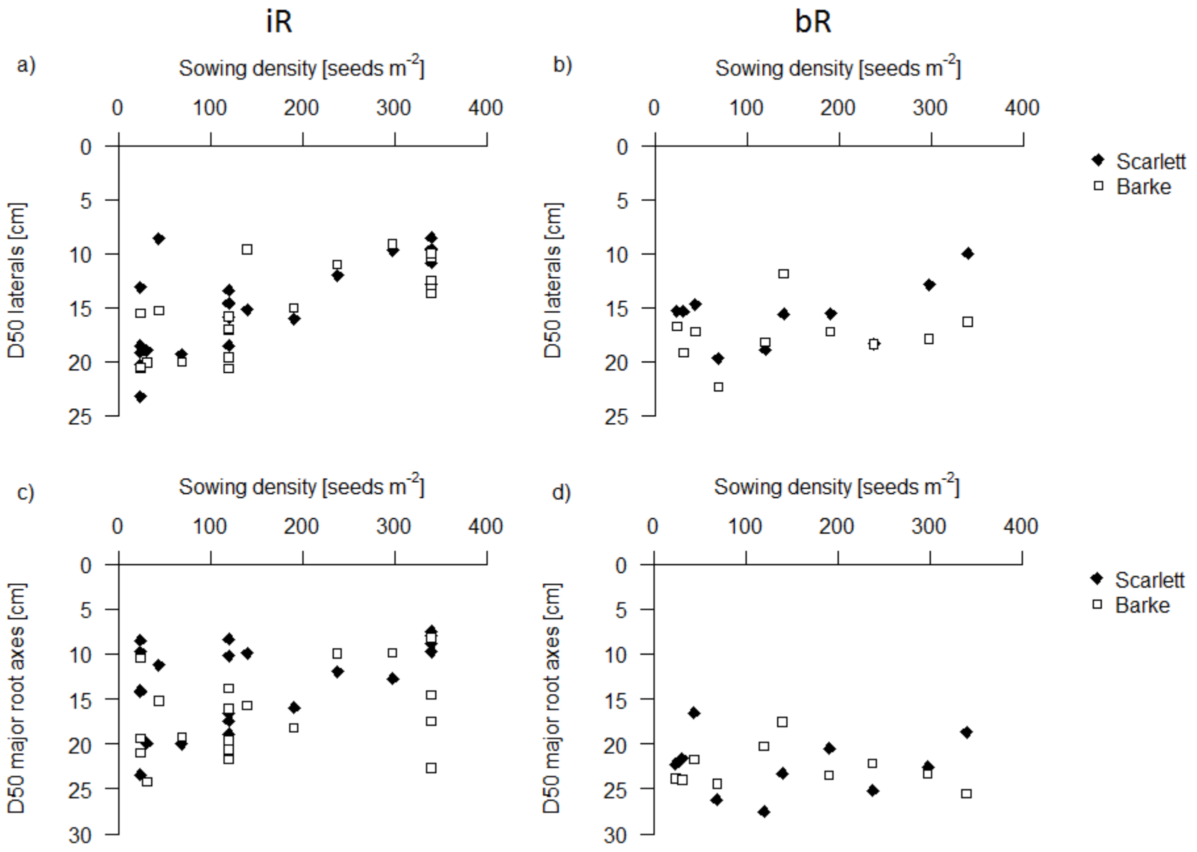

1

Figure S11 | D50 values of laterals (top) and major root axes (bottom) in 2013 in the row (iR) (left) and between the rows (bR) (right) for Scarlett (Sca) and Barke (Bar) calculated over 40 cm. Data are raw data.

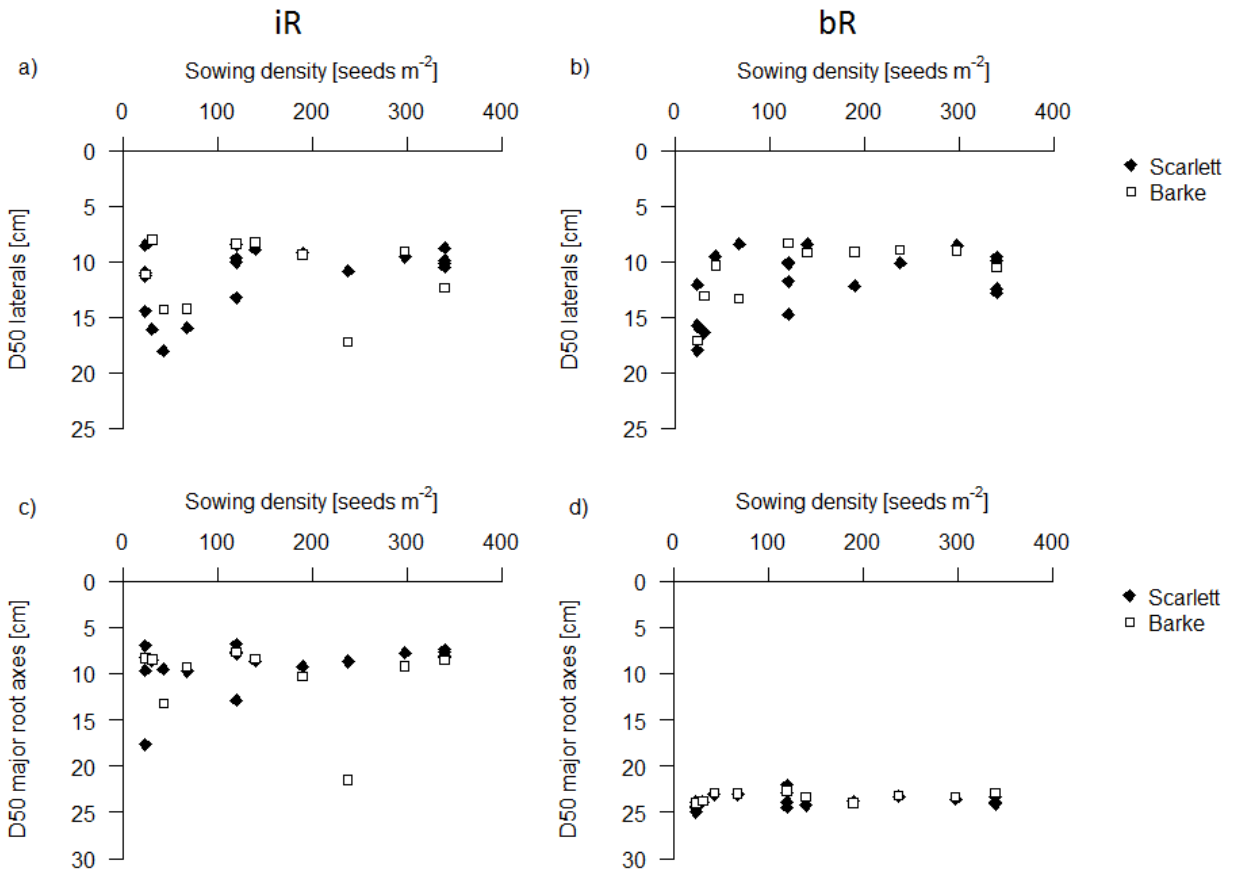

Figure S12 | D50 values of laterals (top) and major root axes (bottom) in 2013 in the row (iR) (left) and between the rows (bR) (right) for Scarlett (Sca) and Barke (Bar) calculated over 40 cm. Data are raw data.
